# Post-recovery health domain scores among outpatients by SARS-CoV-2 testing status during the pre-Delta period

**DOI:** 10.1186/s12879-024-09108-3

**Published:** 2024-03-08

**Authors:** Jennifer P. King, Jessie R. Chung, James G. Donahue, Emily T. Martin, Aleda M. Leis, Arnold S. Monto, Manjusha Gaglani, Kayan Dunnigan, Chandni Raiyani, Sharon Saydah, Brendan Flannery, Edward A. Belongia

**Affiliations:** 1 Center for Clinical Epidemiology and Population Health, Marshfield Clinic Research Institute, 1000 North Oak Avenue ML2, Marshfield, WI 54449 USA; 2 Influenza Division, US Centers for Disease Control and Prevention, 1600 Clifton Rd, Mailstop H24-7, Atlanta, GA 30329 USA; 3 Department of Epidemiology, University of Michigan School of Public Health, Ann Arbor, MI USA; 4 Baylor Scott & White Health, Temple, TX USA; 5 Texas A&M University College of Medicine, Temple, TX USA; 6 Baylor Scott & White Research Institute, Temple, TX USA; 7 Coronavirus and Other Respiratory Viruses Division, US Centers for Disease Control and Prevention, Atlanta, GA USA

**Keywords:** COVID-19, Long COVID, Post-COVID-19 condition

## Abstract

**Background:**

Symptoms of COVID-19 including fatigue and dyspnea, may persist for weeks to months after SARS-CoV-2 infection. This study compared self-reported disability among SARS-CoV-2-positive and negative persons with mild to moderate COVID-19-like illness who presented for outpatient care before widespread COVID-19 vaccination.

**Methods:**

Unvaccinated adults with COVID-19-like illness enrolled within 10 days of illness onset at three US Flu Vaccine Effectiveness Network sites were tested for SARS-CoV-2 by molecular assay. Enrollees completed an enrollment questionnaire and two follow-up surveys (7–24 days and 2–7 months after illness onset) online or by phone to assess illness characteristics and health status. The second follow-up survey included questions measuring global health, physical function, fatigue, and dyspnea. Scores in the four domains were compared by participants’ SARS-CoV-2 test results in univariate analysis and multivariable Gamma regression.

**Results:**

During September 22, 2020 – February 13, 2021, 2712 eligible adults were enrolled, 1541 completed the first follow-up survey, and 650 completed the second follow-up survey. SARS-CoV-2-positive participants were more likely to report fever at acute illness but were otherwise comparable to SARS-CoV-2-negative participants. At first follow-up, SARS-CoV-2-positive participants were less likely to have reported fully or mostly recovered from their illness compared to SARS-CoV-2-negative participants. At second follow-up, no differences by SARS-CoV-2 test results were detected in the four domains in the multivariable model.

**Conclusion:**

Self-reported disability was similar among outpatient SARS-CoV-2-positive and -negative adults 2–7 months after illness onset.

**Supplementary Information:**

The online version contains supplementary material available at 10.1186/s12879-024-09108-3.

## Background

The clinical syndrome of COVID-19 is characterized by a dry cough, fever, and dyspnea [1, 2]. Additional symptoms including, headache, muscle aches, sore throat, and diarrhea are frequently reported [3]. Intermediate and long-term effects of infection with SARS-CoV-2 are recognized as a spectrum of post-COVID-19 condition (PCC), also referred to as Long COVID [4–7]. Symptoms including fatigue and dyspnea may persist for weeks to months even in persons with mild-to-moderate acute illness [8–10]. Evidence suggests that SARS-CoV-2 may cause long-lasting or permanent damage to the lungs and other organ systems following infection, as has been seen with SARS [11, 12]. The burden of PCC in the United States is substantial; by November 2021, among more than 46 million US adults estimated to have had COVID-19, 3–5 million experienced activity-limiting PCC [13, 14]. More recent surveys report that 11.2% of US adults who have ever had COVID-19 report PCC [15].

This study employed four validated measures of global health, fatigue, physical function, and dyspnea to assess the level of self-reported disability 2–7 months after acute COVID-19 and compare disability between participants with COVID-19 and non-COVID-19 illness based on SARS-CoV-2 testing. The study was conducted September 2020 – February 2021 before widespread COVID-19 vaccination when mask mandates for public spaces were still in effect and there were restrictions in place related to restaurants, bars, and schools [16].

## Methods

The source population for this study was adults (≥18 years of age) enrolled in a descriptive study of COVID-19 epidemiology at US Influenza Vaccine Effectiveness (Flu VE) network study sites in Michigan, Wisconsin, and Texas. We enrolled symptomatic persons seeking outpatient medical care (i.e., telehealth, primary care, urgent care, and emergency departments) or testing for SARS-CoV-2. All participants had COVID-19-like illness defined as acute respiratory illness that included fever, cough, or loss of taste or smell. Respiratory specimens (nasal or nasopharyngeal) collected by healthcare providers or study staff within 10 days of illness onset were tested for SARS-CoV-2 using molecular assays as previously described [17]; results were used to classify participants as having COVID-19 or non-COVID-19 illness. COVID-19 vaccination status and presence of underlying health conditions prior to illness onset were extracted from the electronic medical record for all participants.

An enrollment questionnaire administered by research staff either in person or by phone collected pre-specified symptoms (shortness of breath/difficulty breathing, nasal congestion, chills, muscle aches, headache, vomiting, diarrhea, or abdominal pain), self-reported general health status, and demographic information. Two follow-up surveys were administered by email or over the phone with research staff. All participants were invited to complete the first follow-up survey approximately 7–24 days after illness onset [18]. The first survey included questions regarding self-reported symptoms, recovery date, if applicable, additional medical care required for the illness, and work productivity (Supplemental Table 1). Participants were aware of their SARS-CoV-2 status by the time of first follow-up survey initiation.

All participants who tested positive for SARS-CoV-2 at enrollment and a random sample of participants who tested negative were invited to complete the second follow-up survey approximately 2–7 months after illness onset. Roll out of the second follow-up survey varied by site and included participants enrolled from September 22, 2020 through February 13, 2021. To estimate the proportion of test-negative participants who became infected with SARS-CoV-2 before completion of the second follow-up survey, SARS-CoV-2 test-negative participants at one site were asked to provide a blood sample at the time of the second survey (28–42 days after illness onset) to test for anti-spike protein SARS-CoV-2 IgG antibodies (Beckman Coulter, Inc. Access SARS-CoV-2 IgG [19]).

The second follow-up survey assessed global health, fatigue, physical function and dyspnea using validated, standardized short form instruments from the Patient-Reported Outcomes Measurement Information System (PROMIS) network (https://www.healthmeasures.net/explore-measurement-systems/promis) (Supplemental Table 2). The items in each instrument measure responses using a 5-point Likert-type scale. PROMIS short form instruments are scored using item-level calibrations and response pattern scoring which is considered more accurate than the use of raw scores. PROMIS uses a T-score–standardized metric in which 50 is the mean T-score of a relevant reference population (i.e., the US general population) and the standard deviation (SD) is 10. For PROMIS measures, higher scores represent a greater degree of the outcome being assessed (e.g., more fatigue). Formal studies of the various instruments in selected patient populations have determined that the short form instruments provided valid results [20–26].

### Primary analysis

We stratified demographic characteristics, self-reported signs/symptoms at enrollment and other characteristics by SARS-CoV-2 test status. Participant characteristics were compared by χ^2^ tests, 2-sample t-tests, or Wilcoxon two-sample tests as appropriate. Standardized scores for global health, fatigue, physical function, and dyspnea were computed and compared by SARS-CoV-2 test results. Gamma regression was used to perform unadjusted and adjusted comparisons of mean T-scores as ratios (i.e., mean T-score among SARS-CoV-2 test-positive participants divided by mean T-score among SARS-CoV-2 test-negative participants) with corresponding 95% confidence intervals [27]. Separate models were fit for each PROMIS domain with the respective PROMIS T-score as the outcome variable. Participants who self-reported positive SARS-CoV-2 test results after enrollment were excluded. SARS-CoV-2 status (SARS-CoV-2 RT-PCR positive vs. negative at enrollment) was the main exposure variable for primary analyses. The base model was adjusted a priori for age (natural cubic spline with quintile knots), sex, interval between onset and follow-up survey (log-transformed days), and study site. Additional potential confounders were evaluated using likelihood ratio tests. Adjustment for health-related factors included covariates for presence of any underlying chronic condition, self-reported cigarette smoking, body mass index, and self-rated general health status. Adjustment for socio-demographic factors included covariates for self-reported race and Hispanic ethnicity, and education level, along with receipt of 2020–21 seasonal influenza vaccine. An additional analysis was conducted on the subgroup of participants from one site for whom blood specimens during follow-up were collected; we excluded participants who had a negative RT-PCR result for SARS-CoV-2 at enrollment but who tested SARS-CoV-2 seropositive 28–42 days after the enrollment illness onset.

Participants were excluded if they did not complete all PROMIS instruments, had uninterpretable SARS-CoV-2 test results at enrollment or if they received ≥1 dose of any COVID-19 vaccine before the enrollment illness onset (Supplemental Fig. 1). All analyses were conducted using SAS 9.4 (SAS Institute, Cary NC). Figures were generated using R 4.0.0 with the ggplot2 package.

### Sensitivity analyses

We conducted three additional analyses to assess the robustness of our primary findings. First, we restricted the analysis to participants who reported fever at enrollment. Second, we restricted the analysis to participants with underlying conditions in three or more categories. Third, we excluded participants who reported they had fully or mostly recovered at the short-term follow-up assessment.

### Sample size

A total of 200 SARS-CoV-2 test-positive and 200 SARS-CoV-2 test-negative participants, would allow detection of an effect size (differences in mean T-scores) of 2.8 with 80% power. This corresponds to a standardized effect size ([group_1_ mean – group_2_ mean]/[SD]) of 0.28 which is generally considered small to moderate in size. Other parameters in the calculation were α = 0.05 and SD of the outcome in the population = 10.

## Results

Between September 22, 2020 and February 13, 2021, 2712 adults were enrolled at the three sites. Of those, 1541 (57%) completed the first follow-up survey. Of 1350 participants invited to complete the second survey, 650 (48%) attempted it, and 578 (89%) were included in this analysis, including 312 participants in the SARS-CoV-2 test-positive group and 266 participants in the SARS-CoV-2 test-negative group. None of the participants tested positive for influenza at enrollment. Participants were enrolled a median of 9 days after illness onset (interquartile range (IQR), 6–14). SARS-CoV-2 test-positive participants reported a mean of 4.5 (SD, 1.6) signs/symptoms at enrollment compared to a mean of 3.8 (SD, 1.6) among SARS-CoV-2 test-negative participants (Table 1). We observed differences in the signs/symptoms reported by participants by SARS-CoV-2 test result. Of note, a greater proportion of SARS-CoV-2-positive participants reported loss of sense of taste/smell compared to SARS-CoV-2 test-negative participants (65% vs 21%, *p* < 0.01). Among other differences, a greater proportion (74%) of participants in the SARS-CoV-2 test-positive group reported fever or chills compared to participants in the SARS-CoV-2 test-negative group (65%) (*p* = 0.02).

**Table 1:** Illness characteristics among participants in the SARS-CoV-2-positive and SARS-CoV-2-negative groups, N (%)

	Positive SARS-CoV-2 result		Negative SARS-CoV-2 result		
	N	%	N	%	*p*-value
N	312	100	266	100	
Total number of symptoms, mean (SD)	4.5	1.6	3.8	1.6	< 0.01
Reported symptoms at enrollment					
Any respiratory symptom	309	99	242	91	< 0.01
Cough	258	83	201	76	0.03
Loss of taste or smell	202	65	57	21	< 0.01
Shortness of breath	124	40	82	31	0.02
Congestion/runny nose^a^	212	86	164	74	< 0.01
Sore throat	157	50	158	59	0.03
Any generalized sign or symptom	300	96	244	92	0.01
Fever or chills	231	74	173	65	0.02
Fatigue	209	67	178	67	0.21
Muscle aches	220	71	140	53	< 0.01
Headache	247	79	182	68	< 0.01
Any gastrointestinal symptom	166	53	126	47	0.15
Nausea/vomiting	84	27	87	33	0.13
Diarrhea	136	44	82	31	< 0.01
Sought subsequent medical care for illness^b^	16	6	18	8	0.51
Reported recovery at 1st follow up survey^c^	197	80	197	90	< 0.01

*SD* standard deviation

^a^Not assessed in 111 participants including 66 SARS-CoV-2 test-positive participants and 164 SARS-CoV-2 test-negative participants

^b^Whether the participant sought medical care for their illness after enrollment as reported at the first follow-up survey. Missing for 41 SARS-CoV-2 test-negative participants and 63 SARS-CoV-2 test-positive participants. Among SARS-CoV-2-positive participants who sought additional care, 3 reported care in emergency department/hospital setting compared to 2 among test-negative participants

^c^Participants who responded “yes” to the question “Have you fully or mostly recovered from your illness?” on the first follow-up survey. Missing for 46 SARS-CoV-2 test-negative participants and 66 SARS-CoV-2 test-positive participants

The median time from illness onset to first follow-up survey was 21 days (IQR 14–30). Participants in the SARS-CoV-2 test-positive group had a shorter interval between onset and first follow-up survey completion (median 16.5 days, IQR 14–26) than participants in the SARS-CoV-2 test-negative group (median 26 days, IQR 16–33) (*p* < 0.01). On the first follow-up survey, 80% of participants in the SARS-CoV-2 test-positive group reported they had mostly or fully recovered from their illness compared to 90% of participants in the SARS-CoV-2 test-negative group. The median reported time between illness onset and date of recovery was 12 days (IQR 10–17) among participants in the SARS-CoV-2 test-positive group compared to 9 days (IQR 5–13) among participants in the SARS-CoV-2 test-negative group. Among participants who reported they had not yet recovered from the acute illness on the first follow-up survey, 19 (73%) participants who tested SARS-CoV-2 positive reported on-going fatigue compared to 7 (54%) participants who tested SARS-CoV-2 negative (*p* = 0.23). Reporting of fever or chills was the same (15%) for both groups.

Among participants who completed the second follow-up survey, mean age was 47.2 years (SD 14.7); participants in the SARS-CoV-2 test-negative group were more likely to be female, a self-reported smoker, vaccinated against influenza, have a higher education level, and were slightly younger (mean age 45.7 years, SD 14.7) than participants in the SARS-CoV-2 test-positive group (mean age 48.5 years, SD 14.6) (*p* = 0.02) (Table 2). Participants completed the second follow-up survey approximately 3 months (median 89 days, IQR 72–111) after illness onset. Participants in the SARS-CoV-2 test-positive group had a shorter interval between onset and second follow-up survey completion (median 89 days, IQR 74–119) than participants in the SARS-CoV-2 test-negative group (median 104 days, IQR 71–125) (*p* < 0.01).

**Table 2:** Demographic and other characteristics of SARS-CoV-2 test-positive and test-negative participants who completed second follow-up survey

	Total		Positive SARS-CoV-2 result		Negative SARS-CoV-2 result		*P*-value^1^
	N^a^	%^a^	N^a^	%^a^	N^a^	%^a^	
N	578	100	312	100	266	100	
Study site							< 0.01
Michigan	277	48	107	34	170	64	
Texas	111	19	66	21	45	17	
Wisconsin	190	33	139	45	51	19	
Female sex^b^	398	69	204	65	194	73	0.04
Age (years)							0.19
Mean (SD)	47.2	14.7	48.5	14.6	45.7	14.7	
18–49	303	52	153	49	150	56	
50–64	203	35	119	38	84	32	
≥ 65	72	12	40	13	32	12	
Race/ethnicity^c^							0.78
White, non-Hispanic	505	87	274	88	231	87	
Black, non-Hispanic	10	2	5	2	5	2	
Other, non-Hispanic	28	5	13	4	15	6	
Hispanic, any race	32	6	19	6	13	5	
Body Mass Index^d^							0.24
Median (IQR)	29.7	25.6–35.1	30.3	25.9–35.4	29.4	24.8–34.7	
Underweight (< 18.5)	2	< 1	1	0.3	1	< 1	
Normal (18.5–24)	115	20	52	17	63	24	
Overweight (25–29)	165	29	91	29	74	28	
Obese (30–39)	201	35	118	38	83	31	
Morbidly Obese (≥40)	73	13	39	13	34	13	
Self-reported underlying health condition^e^	204	35	107	34	97	36	0.55
Documented underlying health condition							
≥ 1 condition	340	59	187	60	153	58	0.58
≥ 3 conditions	123	21	62	20	61	23	0.50
Metabolic disease	156	27	82	26	74	28	
Hypertension	131	23	73	23	58	22	
Chronic pulmonary disease	78	13	40	13	38	14	
Neurological/musculoskeletal	64	11	27	9	37	14	
Endocrine disorder	63	11	38	12	25	9	
Chronic cardiac disease	61	11	32	10	29	11	
Diabetes mellitus	61	11	38	12	23	9	
Malignancy	51	9	25	8	26	10	
Chronic renal disease	31	5	18	6	13	5	
Immunosuppressive disorder	30	5	15	5	15	6	
Other condition^f^	24	4	13	4	11	4	
Liver disease	23	4	12	4	11	4	
Self-rated general health status							< 0.01
Excellent	139	24	73	23	66	25	
Very Good or Good	413	71	233	75	180	68	
Fair or Poor	26	5	6	2	20	8	
Received 2020–21 influenza vaccine^g^	375	65	190	61	185	70	0.02
Cigarette smoking							0.04
Every day or some days	39	7	15	5	24	9	
Not at all	539	93	297	95	242	91	
Education							< 0.01
Less than high school/high school graduate/GED	77	13	52	17	25	10	
Some college^h^	177	31	106	34	71	27	
Bachelor’s degree	191	33	94	30	97	36	
Advanced degree	133	23	60	19	73	27	
Month of illness onset							< 0.01
September 2020	38	7	4	1	34	13	
October 2020	110	19	42	13	68	26	
November 2020	298	52	198	63	100	38	
December 2020	52	9	28	9	24	9	
January 2021	77	13	39	13	38	14	
February 2021	3	1	1	0.9	2	1	
Interval between onset and 2nd follow up, median days (IQR)	89	72–111	89	74–119	104	71–125	< 0.01

*GED* general education degree, *IQR* interquartile range, *SD* standard deviation

^1^ *P*-value for the comparison between SARS-CoV-2 test-positive and test negative participants

^a^Cell numbers are number or column percent unless otherwise specified

^b^Missing for 1 SARS-CoV-2 test-negative participant

^c^Self-reported at enrollment. Other race includes participants who selected Asian, Native Hawaiian/Other Pacific Islander, American Indian/Alaska Native, or Other and participants who selected more than one race. Missing for 2 SARS-CoV-2 test-negative and 1 SARS-CoV-2 test-positive participants

^d^Missing for 11 SARS-CoV-2 test-negative and 11 SARS-CoV-2 test-positive participants

^e^Self-reported at enrollment to have any of the following: heart disease, lung disease, diabetes, cancer, liver or kidney disease, immune suppression, or high blood pressure. Missing for 4 SARS-CoV-2 test-negative and 2 SARS-CoV-2 test-positive participants

^f^Includes hemoglobinopathies, cerebrovascular disease, and disease of arteries, arterioles, and capillaries

^g^Seasonal influenza vaccination receipt as self-reported at enrollment. Missing for 2 SARS-CoV-2 test-negative and 1 SARS-CoV-2 test-positive participants

^h^Includes vocational training or associate degree

**Fig. 1:**
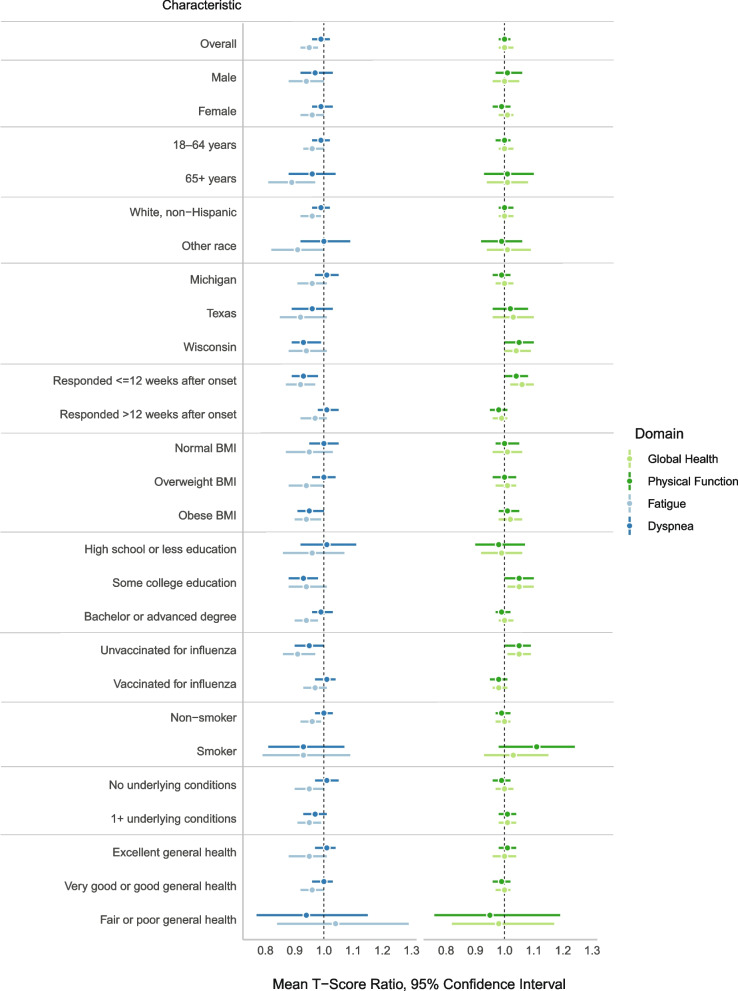
Unadjusted health domain mean T-score ratios^1^ stratified by participant characteristics. ^1^ Defined as mean T-score among SARS-CoV-2 test-positive participants divided by mean T-score among SARS-CoV-2 test-negative participants. For the global health and physical function domains, a mean T-score ratio > 1 indicates better health among SARS-CoV-2 test-positive participants. For the fatigue and dyspnea domains, a mean T-score ratio < 1 indicates better health among SARS-CoV-2 test-positive participants. Higher mean T-score represents more of the concept being measured. For negatively worded questions, a mean T-score of 60 is one SD worse than average; a mean T-score of 40 is one SD better than average. For the physical function and global health domains, a higher mean T-score corresponds to better health. For the dyspnea and fatigue domains, a higher mean T-score corresponds to greater limitation or more fatigue

At the second follow-up time point, there was no overall difference between the groups in unadjusted mean T-scores in the domains of global health, physical function, or dyspnea; however, participants in the SARS-CoV-2 test-negative group reported more fatigue than participants in the SARS-CoV-2 test-positive group (Fig. 1, Supplemental Table 3). Among participants in the SARS-CoV-2 test-negative group, unadjusted mean T-scores (SD) for the global health, physical function, fatigue, and dyspnea domains were 51.7 (7.4), 52.2 (7.1), 46.9 (10.2), and 40.6 (7.9), respectively. Among participants in the SARS-CoV-2 test-positive group, mean T-scores (SD) for the global health, physical function, fatigue, and dyspnea domains were 51.9 (7.2), 52.1 (7.2), 44.6 (9.4), and 40.1 (7.1), respectively.

We observed statistically significant mean T-score ratios within subgroups of participants (e.g., among those who completed the second follow-up survey within 3 months after illness onset), which was generally in the same direction within a domain. Among participants who completed the second follow-up survey within 3 months of illness onset, participants in the SARS-CoV-2 test-negative group reported more impairment on their health and function compared to participants in the SARS-CoV-2 test-positive group. Differences were not observed among participants who completed the second follow-up survey more than 3 months after illness onset.

**Fig. 2:**
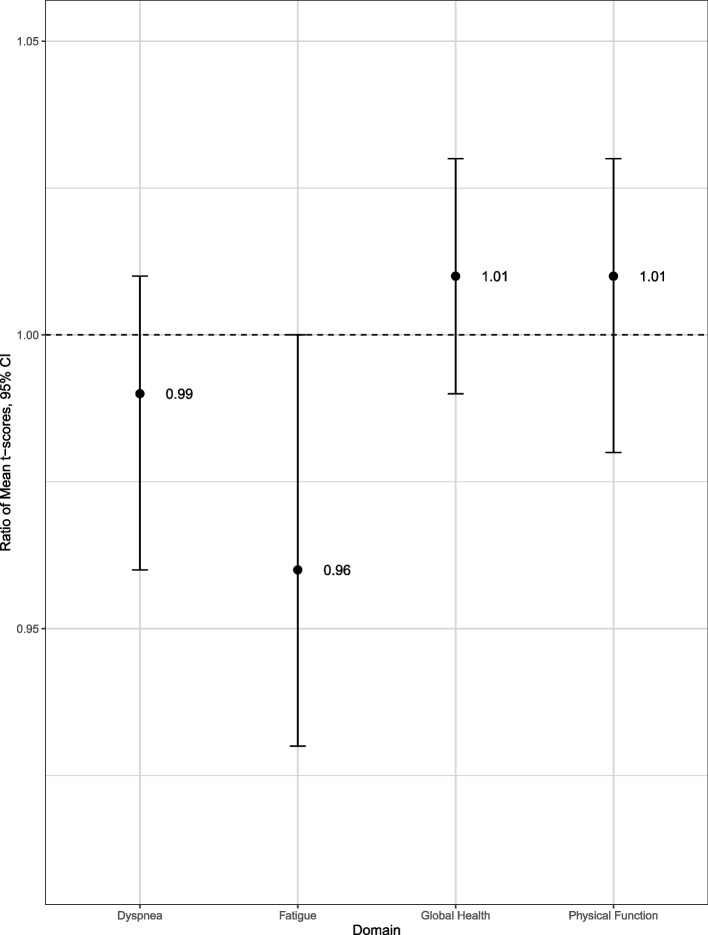
Adjusted health domain mean T-score ratios from multivariable^1^ Gamma regression

After adjustment for participant age at enrollment, participant sex, interval between onset and 2nd follow-up survey completion, study site, presence of any underlying health condition, participant-reported cigarette smoking, and self-rated general health status, we observed no statistically significant mean T-score ratios in any domain (Fig. 2). The addition of social factors to the model including base and health factors did not improve model fit or change interpretation of findings (Supplemental Fig. 2).

Among 51 SARS-CoV-2 test-negative participants from the Wisconsin site who had SARS-CoV-2 serologic testing, 5 (10%) tested seropositive at second follow-up that occurred during the same pre-Delta variant period. These participants were similar to other participants in the SARS-CoV-2 test-negative group with respect to age, sex, and month of illness onset; reported symptoms at enrollment among the five SARS-CoV-2 seropositive participants included cough (*n* = 5), fever (*n* = 3), and sore throat (*n* = 2). Adjusted mean T-score ratios were similar for all domains, when the five participants who were seropositive in the SARS-CoV-2 test-negative group were excluded (Supplemental Table 4).

Adjusted mean T-score ratios in the three sensitivity analyses were generally consistent with primary analyses (Supplemental Table 4). In the sensitivity analysis restricted to participants with underlying conditions in three or more categories, SARS-CoV-2 test-positive participants tended to experience worse outcomes for physical function and fatigue but these differences were not statistically significant.

## Discussion

In this observational study of adults seeking outpatient medical care for an acute symptomatic respiratory illness from September 2020 through February 2021 when COVID-19 vaccines were not yet widely available, there was no difference between SARS-CoV-2 test-positive compared to SARS-CoV-2 test-negative participants surveyed 2–7 months after illness onset in self-reported global health, fatigue, physical function, or dyspnea as measured using four validated PROMIS domains. These findings contribute to evidence that prevalence of symptoms and conditions consistent with PCC may not be limited to post-SARS-CoV-2 respiratory viral infection [28]. This study had several strengths including systematic testing to confirm SARS-CoV-2 status, a geographically diverse study population, and the use of standardized instruments. The examination of prevalence of symptoms following SARS-CoV-2 infection when vaccines were not widely available provides additional evidence to the importance of vaccination in preventing PCC. While PCC continues to occur among those infected after vaccination, the risk is diminished [10, 28, 29].

The prevalence of persistent symptoms more than 2 months after illness onset and overall effects on well-being following acute illness found in this study are within the range reported from other studies following patients diagnosed with medically attended COVID-19 or mildly symptomatic SARS-CoV-2 infection [30–32]. Three similar studies, including one that also measured specific health domains, reported comparable results when SARS-CoV-2 test-positive patients were compared to SARS-CoV-2 test-negative patients [33–35]. While acute symptoms and quality of life indicators may differ between ambulatory patients with and without SARS-CoV-2, there is substantial overlap in the clinical features of infection caused by SARS-CoV-2 and other respiratory viruses, such as influenza [17, 36]. Participants in both groups in this analysis were more likely to report being fully or mostly recovered from their illness on the first follow-up survey compared to what was reported among adults enrolled in the US Flu Vaccine Effectiveness Network during the 2017–18 influenza season. In that pre-COVID-19 pandemic influenza season when approximately one-third of participants tested positive for influenza, 32% of adults aged 19–64 years who completed the follow-up survey reported they had not yet fully or mostly recovered 7–21 days after illness onset [37]. In that influenza season, the median time between illness onset and recovery among participants who had fully or mostly recovered at follow-up was 11 days, similar to median duration of illness observed in this study (9 days for SARS-CoV-2 test-negative and 12 days for SARS-CoV-2 test-positive participants).

It remains unclear how prevalent long-term sequelae are with respect to other common respiratory viral pathogens and why SARS-CoV-2 infection can result in long-term sequelae in some individuals but not in others. Introduction of zoonotic coronavirus infection in naïve human populations with limited cross-protection from common human coronaviruses may have increased pathogenicity or intensity of human immune response until SARS-CoV-2 adapted to human hosts and the population developed partial immunity. Alternatively, the magnitude of COVID-19 cases may have increased attention to post-viral syndromes and persistence of symptoms common to many viral infections, including fatigue and persistent decrease in lung function. Early recognition of persistent or new symptoms, including fatigue, months after laboratory-confirmed COVID-19 may have increased awareness of and healthcare seeking for PCC [38]. Many early reports and studies described more severe post-COVID-19 syndromes following severe and prolonged acute illness [39, 40]. The description of and evidence for less severe PCC following even mild symptomatic COVID-19 followed from cohort studies and suggested that most mild illness was self-limited. Findings were similar in the sensitivity analysis in this study that was restricted to outpatients who reported fever. Because persistent symptoms occur following many viral infections and infections may exacerbate underlying chronic conditions with similar clinical presentation, inclusion of a comparison group of patients with mild illness who test negative for acute SARS-CoV-2 infection is needed to identify specific characteristics of post-SARS-CoV-2 infection sequelae.

The findings presented here are subject to several limitations. First, our findings may not be generalizable to the wider population of adults who might seek care for mild to moderate COVID-19 in the present era. During the period of this study, there could have been differences in the people who were seeking care due to restrictions on in-person medical encounters or the potential for fear of social stigma related to testing positive for SARS-CoV-2. During this period, mask mandates for public spaces were still in effect in these states, there were bans on indoor dining, and Texas and Wisconsin were still under Emergency Orders [16]. Persons who presented for care during this time might have had more underlying medical conditions or other unmeasured differences. The proportion of participants with an underlying condition who completed the second survey (59%, Table 2) was greater than what was observed among adults enrolled in the US Flu VE Network over multiple seasons prior to the COVID-19 pandemic [41]. Further, many patients approached for participation in the follow-up surveys following initial SARS-CoV-2 test declined, leading to a non-representative and potentially biased sample of all symptomatic patients. Second, we did not test controls for other etiologies besides SARS-CoV-2 and influenza viruses. Finally, our comparison group may have been previously infected with SARS-CoV-2 or infected before the follow-up surveys after testing negative for infection at acute illness. Serology conducted only at one site indicated that approximately 10% of SARS-CoV-2 test-negative patients enrolled at that site had been infected before the second follow-up survey. While excluding these patients did not change results, inclusion of patients with SARS-CoV-2 in the comparison group would bias results towards the null.

These results highlight that many individuals may continue to experience ongoing symptoms in the weeks following an acute respiratory infection and that these symptoms are likely not unique to SARS-CoV-2 infection. Characterization of PCC remains challenging as immune and vaccine history grows more complex and with the emergence of new SARS-CoV-2 variants. Our study was conducted during a unique time during the COVID-19 pandemic. Importantly, the study period preceded widespread COVID-19 vaccination and emergence of the Delta and Omicron variants when patients may have had multiple SARS-CoV-2 infections. As a result, the unvaccinated participants who tested SARS-CoV-2 positive at enrollment were likely infected for the first time. Widespread COVID-19 vaccination efforts and booster campaigns may reduce the occurrence of PCC or modify its characteristics [42]. Inclusion of comparison groups of symptomatic patients with medically attended illness in future evaluations of PCC will help identify contributing SARS-CoV-2-specific factors versus non-specific factors that could be targeted with different interventions.

### Supplementary Information

**Additional file 1: Supplemental Table 1.** First follow-up questionnaire questions common to all sites. **Supplemental Table 2**. PROMIS questions administered on second follow-up survey. **Supplemental Table 3**. Unadjusted raw PROMIS mean T-scores^1^ and mean T-score ratios^2^ by SARS-CoV-2 test-positive or SARS-CoV-2 test-negative groups and participant characteristics. **Supplemental Table 4**. Multivariable Gamma regression mean T-score ratios and 95% confidence intervals from subgroup and sensitivity analyses. Models adjusted for base factors and health factors. **Supplemental Figure 1.** Survey completion and participant exclusions. **Supplemental Figure 2.** Adjusted Global Health, Physical Function, Fatigue, and Dyspnea domain mean T-score ratios from multivariable^1-3^ Gamma regression models.

## Data Availability

The datasets analyzed during the current study are not publicly available because they contain personally identifiable information but are available from the corresponding author on reasonable request.
