# Supplementary material for: Post-recovery health domain scores among outpatients by SARS-CoV-2 testing status during the pre-Delta period
Source: BMC Infect Dis. 2024 Mar 8;24:300. doi: 10.1186/s12879-024-09108-3 (PMC10921777; doi:10.1186/s12879-024-09108-3)
Supplement: Supplementary file 1 — Additional file 1: Supplemental Table 1. First follow-up questionnaire questions common to all sites. Supplemental Table 2. PROMIS questions administered on second follow-up survey. Supplemental Table 3. Unadjusted raw PROMIS mean T-scores1 and mean T-score ratios2 by SARS-CoV-2 test-positive or SARS-CoV-2 test-negative groups and participant characteristics. Supplemental Table 4. Multivariable Gamma regression mean T-score ratios and 95% confidence intervals from subgroup and sensitivity analyses. Models adjusted for base factors and health factors. Supplemental Figure 1. Survey completion and participant exclusions. Supplemental Figure 2. Adjusted Global Health, Physical Function, Fatigue, and Dyspnea domain mean T-score ratios from multivariable1-3 Gamma regression models. [file 12879_2024_9108_MOESM1_ESM.docx]

**Supplemental Table 1.** First follow-up questionnaire questions common to all sites

1. Have you returned to your normal activities (ex. go back to work, exercise,
housework/chores)?

__Yes: Date: ___ ___-___ ___-___ ___ ___ ___ (mm/dd/yyyy)

__ I have not yet returned to my normal activities

__ I never stopped doing my normal activities

__ Don’t Know

__ Refused

2. Have you fully or mostly recovered from this illness?

__ Yes: Date: ___ ___-___ ___-___ ___ ___ ___ (mm/dd/yyyy)

__ I have not fully or mostly recovered from this illness

__ Don’t Know

__ Refused

[IF NO] 3. What symptoms have you experienced in the past 24 hours?

| **Symptoms** | | **Yes** | **No** | **Don’t Know** | **Refused** |
| --- | --- | --- | --- | --- | --- |
|  | Fever/Feverishness |  |  |  |  |
|  | Chills |  |  |  |  |
|  | Cough |  |  |  |  |
|  | Sore throat |  |  |  |  |
|  | Difficulty breathing/shortness of breath |  |  |  |  |
|  | Muscle aches |  |  |  |  |
|  | Fatigue/run down |  |  |  |  |
|  | Nausea/vomiting |  |  |  |  |
|  | Diarrhea |  |  |  |  |
|  | Headache |  |  |  |  |
|  | Decreased or complete loss of smell or taste |  |  |  |  |
|  | Other, specify: | | | | |

4. After enrollment in this study, did you seek additional medical care for this recent illness?

__ Yes __ No __ Don’t know __Refused

**[If yes],** how many visits? __ visits

**[If yes],** where did you receive medical care after enrollment? (Please check all that apply)

⬜ Doctor’s office

⬜ Urgent care

⬜ Retail Pharmacy clinic

⬜ Emergency Department/Hospital

⬜ Other, Specify: ___ (i.e., nurseline)

⬜ Refused

**[Additional questions for adults aged 19-64 years]**

**We are studying how respiratory illnesses affect people’s ability to work. The following questions are about your current job and how your recent respiratory illness affected your work. If you work multiple jobs, please think about the job you consider your primary job when answering these questions.**

1. Are you currently employed (work for pay or profit)?
    __ I work for an employer
    __ I am self-employed or own my own business 🡺 [**Survey is complete]**

__ No 🡺 [**Survey is complete]**

__ Refused 🡺 [**Survey is complete]**

1. During the month before illness, how many hours were you expected to work in a week?

(If it varies, estimate the average)

_ _ _Hours __Don’t Know __ Refused

[**If hours is < 20, Don’t Know, Refused, or missing, survey is complete**]

1. Of those expected hours, how many hours in a week did you usually work from home (telework, telecommute, or remote work)? (Enter “0” if none)

_ _ _ Hours __Don’t Know __ Refused

**[if expected hours equals hours worked from home, survey is complete]**

1. Policies for working remotely may differ for jobs that are salaried vs pay hourly. Are you salaried or are you paid hourly? [“Salaried” means you’re paid the same amount each week or month no matter how many hours you work. “Hourly” means that you’re paid a different amount each week or month depending on how many hours you work.]

__ Salaried __ Paid hourly__ Other such as commission only

__ Don’t know __ Refused

1. In an average work week, how much of your time do you spend in direct contact with other people at work? Direct contact means close, face to face interaction within about 3 feet.

__ Less than half of the time

__ About half of the time

__ Most of the time

__ Don’t Know

__ Refused

1. If you miss work because you are sick, do you still get paid (i.e., you have a certain amount of paid sick time that you can use each year)?

__ Yes __ No __Don’t know __Refused

**Please think about the first three days of your illness. The first day of illness being the day your symptoms started [DAY/DATE OF ONSET] and the third day of illness being [DATE OF ONSET + 2].**

1. On the first day of illness [**DATE OF ONSET**]:

11a. How many hours were you scheduled to work? **[Enter number of hours]**

_ _ Hours **🡪 [If Q.11a = 0, skip to Q.12]**

11b. How many hours did you work? **[Enter number of hours]**

_ _ Hours

- - 1. **[IF Q.11b LESS THAN Q.11a OR IS 0]** Did you work less than you were scheduled because of your illness?

__ Yes __ No __ Don’t Know __ Refused

11c. **[IF Q.11b IS > 0]** Where did you work?

__ At work

__ Remotely

__ Both at work and remotely

__ Don’t Know

__ Refused

1. On the second day of illness [**DATE OF ONSET+1**]:

12a. How many hours were you scheduled to work? **[Enter number of hours]**

_ _ Hours 🡪 **[If Q.12a = 0, skip to Q.13]**

12b. How many hours did you work? **[Enter number of hours]**

_ _ Hours

- - 1. **[IF Q.12b < Q.12a or = 0]** Did you work less than you were scheduled because of your illness?

__ Yes __ No __ Don’t Know __ Refused

12c. **[IF Q.12b > 0]** Where did you work?

__ At work

__ Remotely

__ Both at work and remotely

__ Don’t Know

__ Refused

1. On the third day of illness **[DATE OF ONSET+2]**:

13a. How many hours were you scheduled to work? **[Enter number of hours]**

_ _ Hours 🡪 **[If Q.13a = 0, skip to Q.14]**

13b. How many hours did you work? **[Enter number of hours]**

_ _ Hours

- - 1. **[IF Q.13b <Q.13a or =0]** Did you work less than you were scheduled because of your illness?

__ Yes __No __Don’t Know __Refused

13c. **[IF Q.13b > 0]** Where did you work?

__ At work

__ Remotely

__ Both at work and remotely

__ Don’t Know

__ Refused

1. On the day before your illness began [DATE OF ONSET-1]:

14a. How many hours were you scheduled to work [Enter number of hours]

_ _ Hours 🡪 **[If Q.14a = 0, then skip to Q.15]**

14b. How many hours did you work? [Enter number of hours]

_ _ Hours

14c. **[IF Q.14b > 0]** Where did you work?

__ At work

__ Remotely

__ Both at work and remotely

__ Don’t Know

__ Refused

**The following questions relate to your job and the company/organization from which you receive your paycheck. If you work multiple jobs, please think about the job you consider your primary job when answering these questions.**

15. Please select your level of agreement with the following statements about your place of work.

|  | **Strongly Agree** | **Agree** | **Neither Agree nor Disagree** | **Disagree** | **Strongly Disagree** |
| --- | --- | --- | --- | --- | --- |
| Employees are discouraged from coming to work when they have flu-like symptoms. |  |  |  |  |  |
| Employees are encouraged to go home if they have flu-like symptoms at work. |  |  |  |  |  |

16. In which industry do you work? (select one)

__ Agriculture (e.g., crops, livestock)

__ Finance, insurance, or real estate (e.g., bank, insurance agent, real estate agent)

__ Health care (e.g., medical clinic, dental clinic, optometrist, home health, hospital)

__ Manufacturing, including food processing (e.g., equipment, chemical, food products)

__ Retail trade or services (e.g., store, hotel, repair, entertainment, consulting, education)

__ Other

__ Don’t Know

__ Refused

**Supplemental Table 2**. PROMIS questions administered on second follow-up survey.

| **Domain** | **Question** | **Answer choices** |
| --- | --- | --- |
| Global Health | In general, how would you rate your physical health? | 1 – Poor  2 – Fair  3 – Good  4 – Very good  5 – Excellent |
|  | To what extent are you able to carry out your everyday physical activities? | 1 – Not at all  2 – A little  3 – Moderately  4 – Mostly  5 – Completely |
| Physical Function | Are you able to do chores such as vacuuming or yard work? | 1 – Unable to do  2 – With much difficulty  3 – With some difficulty  4 – With a little difficulty  5 – Without any difficulty |
|  | Are you able to go up and down stairs at a normal pace? | 1 – Unable to do  2 – With much difficulty  3 – With some difficulty  4 – With a little difficulty  5 – Without any difficulty |
|  | Are you able to go for a walk of at least 15 minutes? | 1 – Unable to do  2 – With much difficulty  3 – With some difficulty  4 – With a little difficulty  5 – Without any difficulty |
|  | Are you able to run errands and shop? | 1 – Unable to do  2 – With much difficulty  3 – With some difficulty  4 – With a little difficulty  5 – Without any difficulty |
| Dyspnea Functional Limitation | Considering your shortness of breath over the past 7 days, rate the amount of difficulty you had when doing the following activities: |  |
|  | Dressing yourself without help | 0 – No difficulty  1 – A little difficulty  2 – Some difficulty  3 – Much difficulty  NA – I did not do this in the past 7 days |
|  | Walking 50 steps/paces on flat ground at a normal speed without stopping | 0 – No difficulty  1 – A little difficulty  2 – Some difficulty  3 – Much difficulty  NA – I did not do this in the past 7 days |
|  | Walking up 20 stairs (2 flights) without stopping | 0 – No difficulty  1 – A little difficulty  2 – Some difficulty  3 – Much difficulty  NA – I did not do this in the past 7 days |
|  | Preparing meals | 0 – No difficulty  1 – A little difficulty  2 – Some difficulty  3 – Much difficulty  NA – I did not do this in the past 7 days |
|  | Washing dishes | 0 – No difficulty  1 – A little difficulty  2 – Some difficulty  3 – Much difficulty  NA – I did not do this in the past 7 days |
|  | Sweeping or mopping | 0 – No difficulty  1 – A little difficulty  2 – Some difficulty  3 – Much difficulty  NA – I did not do this in the past 7 days |
|  | Making a bed | 0 – No difficulty  1 – A little difficulty  2 – Some difficulty  3 – Much difficulty  NA – I did not do this in the past 7 days |
|  | Lifting something weighing 10-20 lbs (about 4.5-9 kg, like a large bag of groceries) | 0 – No difficulty  1 – A little difficulty  2 – Some difficulty  3 – Much difficulty  NA – I did not do this in the past 7 days |
|  | Carrying something weighing 10-20 lbs (about 4.5-9 kg, like a large bag of groceries) from one room to another | 0 – No difficulty  1 – A little difficulty  2 – Some difficulty  3 – Much difficulty  NA – I did not do this in the past 7 days |
|  | Walking (faster than your usual speed) for ½ mile (almost 1 km) without stopping | 0 – No difficulty  1 – A little difficulty  2 – Some difficulty  3 – Much difficulty  NA – I did not do this in the past 7 days |
| Fatigue | During the past 7 days:  I feel fatigued | 1 – Not at all  2 – A little bit  3 – Somewhat  4 – Quite a bit  5 – Very much |
|  | I have trouble starting things because I am tired | 1 – Not at all  2 – A little bit  3 – Somewhat  4 – Quite a bit  5 – Very much |
|  | In the past 7 days: |  |
|  | How run-down did you feel on average? | 1 – Not at all  2 – A little bit  3 – Somewhat  4 – Quite a bit  5 – Very much |
|  | How fatigued were you on average? | 1 – Not at all  2 – A little bit  3 – Somewhat  4 – Quite a bit  5 – Very much |

NA, not applicable

**Supplemental Table 3**. Unadjusted raw PROMIS mean T-scores^1^ and mean T-score ratios^2^ by SARS-CoV-2 test-positive or SARS-CoV-2 test-negative groups and participant characteristics

|  | **Global health^3^** | | | **Physical function^3^** | | | **Fatigue^4^** | | | **Dyspnea^4^** | | |
| --- | --- | --- | --- | --- | --- | --- | --- | --- | --- | --- | --- | --- |
|  | **SARS-CoV-2 negative** | **SARS-CoV-2 positive** | **Ratio (95% CI)** | **SARS-CoV-2 negative** | **SARS-CoV-2 positive** | **Ratio (95% CI)** | **SARS-CoV-2 negative** | **SARS-CoV-2 positive** | **Ratio**  **(95% CI)** | **SARS-CoV-2 negative** | **SARS-CoV-2 positive** | **Ratio**  **(95% CI)** |
| **Overall** | 51.7 (7.4) | 51.9 (7.2) | 1.00  (0.98-1.03) | 52.2 (7.1) | 52.1 (7.2) | 1.00  (0.98-1.02) | **46.9 (10.2)** | **44.6 (9.4)** | **0.95**  **(0.92-0.98)** | 40.6 (7.9) | 40.1 (7.1) | 0.99  (0.96-1.02) |
| **Sex** | | | | | | | | | | | | |
| Male | 51.2 (8.0) | 51.4 (7.1) | 1.00  (0.96-1.05) | 52.2 (7.3) | 52.9 (7.4) | 1.01  (0.97-1.06) | 46.4 (9.6) | 43.6 (9.4) | 0.94  (0.88-1.00) | 40.6 (8.3) | 39.5 (6.9) | 0.97  (0.92-1.03) |
| Female | 51.8 (7.2) | 52.1 (7.2) | 1.01  (0.98-1.03) | 52.1 (7.0) | 51.7 (7.1) | 0.99  (0.96-1.02) | 47.2 (10.4) | 45.1 (9.4) | 0.96  (0.92-1.00) | 40.6 (7.7) | 40.3 (7.2) | 0.99  (0.96-1.03) |
| **Age group (years)** | | | | | | | | | | | | |
| 18–64 | 51.8 (7.4) | 52.0 (7.2) | 1.00  (0.98-1.03) | 52.6 (6.8) | 52.6 (6.9) | 1.00  (0.97-1.02) | 46.8 (10.4) | 45.0 (9.4) | 0.96  (0.93-1.00) | 40.1 (7.7) | 39.8 (7.0) | 0.99  (0.96-1.02) |
| 65+ | 50.5 (7.1) | 51.0 (7.3) | 1.01  (0.94-1.08) | 49.0 (8.1) | 49.4 (9.0) | 1.01  (0.93-1.10) | **47.6 (8.3)** | **42.2 (9.7)** | **0.89**  **(0.81-0.97)** | 43.8 (8.4) | 41.9 (7.5) | 0.96  (0.88-1.04) |
| **Race/ethnicity** | | | | | | | | | | | | |
| White, non-Hispanic | 52.0 (7.2) | 52.2 (7.1) | 1.00  (0.98-1.03) | 52.3 (7.0) | 52.3 (7.2) | 1.00  (0.98-1.03) | **46.3 (10.0)** | **44.4 (9.5)** | **0.96**  **(0.92-0.99)** | 40.6 (7.9) | 40.0 (7.0) | 0.99  (0.96 -1.02) |
| Other | 49.1 (8.2) | 49.7 (7.6) | 1.01  (0.94-1.09) | 51.6 (7.6) | 50.9 (7.6) | 0.99  (0.92-1.06) | 50.7 (10.9) | 46.0 (9.2) | 0.91  (0.82-1.00) | 40.6 (7.4) | 40.7 (7.8) | 1.00  (0.92-1.09) |
| **Site** | | | | | | | | | | | | |
| Michigan | 53.3 (6.5) | 53.3 (6.2) | 1.00  (0.97-1.03) | 53.5 (6.3) | 53.0 (6.4) | 0.99  (0.96-1.02) | 46.2 (9.6) | 44.3 (9.2) | 0.96  (0.91-1.01) | 39.2 (7.0) | 39.4 (6.7) | 1.01  (0.97-1.05) |
| Texas | 46.7 (7.8) | 48.0 (8.3) | 1.03 (0.96-1.10) | 49.9 (8.2) | 50.7 (7.6) | 1.02  (0.96-1.08) | 49.4 (12.0) | 45.5 (10.0) | 0.92  (0.85-1.01) | 43.4 (10.0) | 41.5 (8.0) | 0.96  (0.89-1.03) |
| Wisconsin | **50.5 (7.6)** | **52.6 (6.7)** | **1.04**  **(1.00-1.09)** | **49.8 (7.5)** | **52.2 (7.6)** | **1.05 (1.00-1.10)** | 47.0 (10.2) | 44.4 (9.4) | 0.94 (0.88-1.01) | **42.7 (7.3)** | **39.9 (7.0)** | **0.93**  **(0.89-0.99)** |
| **Time from Onset** | | | | | | | | | | | | |
| ≤12 weeks | **48.4 (7.8)** | **51.3 (7.5)** | **1.06**  **(1.02-1.10)** | **50.0 (7.8)** | **52.1 (6.9)** | **1.04**  **(1.00-1.08)** | **48.6 (11.1)** | **44.7 (9.2)** | **0.92**  **(0.87-0.97)** | **43.0 (8.8)** | **40.2 (7.1)** | **0.93**  **(0.89-0.98)** |
| >12 weeks | 53.3 (6.6) | 52.6 (6.7) | 0.99  (0.96-1.01) | 53.3 (6.4) | 52.2 (7.6) | 0.98  (0.95-1.01) | 46.0 (9.6) | 44.5 (9.8) | 0.97  (0.92-1.01) | 39.3 (7.0) | 39.8 (7.2) | 1.01  (0.98-1.05) |
| **Body Mass Index** | | | | | | | | | | | | |
| Normal | 55.1 (6.9) | 55.4 (7.0) | 1.01  (0.96-1.06) | 54.4 (5.5) | 54.7 (5.3) | 1.00  (0.97-1.05) | 45.1 (11.4) | 42.8 (9.0) | 0.95  (0.87-1.03) | 37.4 (5.8) | 37.4 (5.2) | 1.00  (0.95-1.05) |
| Overweight | 53.4 (5.7) | 53.9 (6.0) | 1.01  (0.97-1.04) | 54.2 (5.6) | 54.3 (6.0) | 1.00  (0.96-1.04) | 45.4 (9.3) | 42.6 (9.5) | 0.94  (0.88-1.00) | 38.2 (5.9) | 38.2 (5.5) | 1.00  (0.96-1.04) |
| Obese | 48.4 (7.5) | 49.4 (7.0) | 1.02  (0.98-1.06) | 49.5 (7.9) | 50.2 (7.8) | 1.01  (0.98-1.05) | **49.2 (9.4)** | **46.5 (9.5)** | **0.94**  **(0.90-0.99)** | **44.1 (8.8)** | **42.0 (7.8)** | **0.95**  **(0.91-1.00)** |
| **Education** | | | | | | | | | | | | |
| High school or less | 49.6 (6.4) | 48.9 (7.8) | 0.99  (0.92-1.06) | 50.6 (7.8) | 49.5 (8.5) | 0.98  (0.90-1.07) | 47.3 (11.9) | 45.4 (10.2) | 0.96  (0.86-1.07) | 41.9 (7.4) | 42.4 (8.6) | 1.01  (0.92-1.11) |
| Some college | **48.1 (8.0)** | **50.7 (6.9)** | **1.05**  **(1.01-1.10)** | **49.4 (8.3)** | **51.8 (7.4)** | **1.05**  **(1.00-1.10)** | 49.2 (11.7) | 46.3 (9.8) | 0.94  (0.88-1.01) | **44.0 (9.2)** | **40.8 (7.4)** | **0.93**  **(0.88-0.98)** |
| Bachelor or Advanced Degree | 53.4 (6.7) | 53.6 (6.7) | 1.00  (0.98-1.03) | 53.6 (6.0) | 53.3 (6.4) | 0.99  (0.97-1.02) | **45.9 (9.1)** | **43.2 (8.8)** | **0.94**  **(0.90-0.98)** | 38.9 (6.8) | 38.7 (6.1) | 0.99  (0.96-1.03) |
| **Flu Vaccine** | | | | | | | | | | | | |
| Unvaccinated | **49.5 (7.8)** | **52.0 (6.4)** | **1.05**  **(1.01-1.09)** | **50.7 (7.9)** | **53.1 (6.5)** | **1.05**  **(1.00-1.09)** | **48.9 (10.3)** | **44.6 (9.4)** | **0.91**  **(0.86-0.97)** | 41.7 (9.2) | 39.5 (6.7) | 0.95  (0.90-1.00) |
| Vaccinated | 52.6 (7.0) | 51.8 (7.6) | 0.98  (0.96-1.01) | 52.9 (6.6) | 51.6 (7.6) | 0.98  (0.95-1.01) | 46.0 (10.1) | 44.6 (9.5) | 0.97  (0.93-1.01) | 40.0 (7.2) | 40.3 (7.3) | 1.01  (0.97-1.04) |
| **Smoking** | | | | | | | | | | | | |
| Non-Smoker | 52.1 (7.2) | 52.0 (7.2) | 1.00  (0.97-1.02) | 52.6 (6.7) | 52.1 (7.3) | 0.99  (0.97-1.02) | **46.4 (9.8)** | **44.4 (9.4)** | **0.96**  **(0.92-0.99)** | 40.1 (7.4) | 39.9 (7.1) | 1.00  (0.97-1.03) |
| Somedays/ Everyday | 47.0 (7.9) | 48.5 (5.8) | 1.03  (0.93-1.15) | 48.1 (9.3) | 53.1 (6.6) | 1.11  (0.98-1.24) | 51.7 (13.1) | 48.0 (10.4) | 0.93  (0.79-1.09) | 45.5 (10.3) | 42.4 (8.2) | 0.93  (0.81-1.07) |
| **Underlying Conditions** | | | | | | | | | | | | |
| None | 53.8 (6.3) | 53.7 (6.7) | 1.00  (0.97-1.03) | 53.9 (5.3) | 53.5 (6.1) | 0.99  (0.96-1.02) | 45.5 (10.4) | 43.4 (8.4) | 0.95  (0.90-1.00) | 38.5 (6.2) | 38.8 (6.3) | 1.01  (0.97-1.05) |
| 1 or more | 50.0 (7.7) | 50.6 (7.2) | 1.01  (0.98-1.04) | 50.9 (7.9) | 51.2 (7.8) | 1.01  (0.98-1.04) | **48.0 (9.9)** | **45.4 (10.0)** | **0.95**  **(0.91-0.99)** | 42.1 (8.6) | 40.8 (7.5) | 0.97  (0.93-1.01) |
| **Self-reported general health status** | | | | | | | | | | | | |
| Excellent | 57.1 (6.0) | 57.1 (6.6) | 1.00  (0.96-1.04) | 54.9 (5.0) | 55.3 (4.4) | 1.01  (0.98-1.04) | 44.2 (10.4) | 41.8 (7.8) | 0.95  (0.88-1.01) | 36.7 (4.7) | 36.8 (4.7) | 1.01  (0.97-1.04) |
| Very good or Good | 50.7 (6.3) | 50.5 (6.4) | 1.00  (0.97-1.02) | 51.9 (6.9) | 51.4 (7.4) | 0.99  (0.96-1.02) | 47.0 (9.4) | 45.1 (9.5) | 0.96  (0.92-1.00) | 41.2 (7.8) | 41.0 (7.4) | 1.00  (0.96-1.03) |
| Fair or Poor | 42.2 (7.5) | 41.3 (7.2) | 0.98  (0.82-1.17) | 46.0 (10.0) | 43.7 (12.6) | 0.95  (0.76-1.19) | 55.0 (12.7) | 57.4 (12.9) | 1.04  (0.84-1.29) | 47.9 (10.0) | 45.1 (7.0) | 0.94  (0.77-1.15) |

CI, confidence interval; SD, standard deviation

^1^ Higher T-score represents more of the concept being measured. For negatively worded questions, a T-score of 60 is one standard deviation worse than average, and T-score of 40 is one standard deviation better than average.

^2^ Calculated as mean T-score for participants in the SARS-CoV-2 test-positive group divided by mean T-score among participants in the SARS-CoV-2 test-negative group. Statistically significant mean T-score ratios are highlighted in bold text.

^3^ Higher score corresponds to better health

^4^ Higher score corresponds to greater limitation or more fatigue.

**Supplemental Table 4**. Multivariable Gamma regression mean T-score ratios and 95% confidence intervals from subgroup and sensitivity analyses. Models adjusted for base factors and health factors.

|  | **Positive SARS-CoV-2 test result (N)** | **Negative SARS-CoV-2 test result (N)** | **Global Health** | **Physical Function** | **Fatigue** | **Dyspnea** |
| --- | --- | --- | --- | --- | --- | --- |
| Primary analysis | 312 | 266 | 1.01 (0.99, 1.03) | 1.01 (0.98, 1.03) | 0.96 (0.93, 1.00) | 0.99 (0.96, 1.01) |
| Participants who reported fever at enrollment | 208 | 132 | 0.99 (0.96, 1.01) | 1.00 (0.97, 1.03) | 0.98 (0.93, 1.03) | 0.99 (0.95, 1.02) |
| Participants with ≥3 underlying health conditions | 62 | 61 | 1.00 (0.95, 1.06) | 0.99 (0.93, 1.06) | 1.01 (0.94, 1.09) | 0.97 (0.90, 1.04) |
| Seropositive participants^1^ excluded | 138 | 46 | 1.02 (0.98, 1.06) | 1.04 (0.99, 1.09) | 0.97 (0.90, 1.05) | 0.95 (0.89, 1.00) |
| Participants who reported having recovered at first follow-up^2^ excluded | 115 | 69 | 0.99 (0.96, 1.03) | 1.00 (0.95, 1.04) | 0.96 (0.90, 1.03) | 0.99 (0.94, 1.05) |

^1^ Participants at the Wisconsin site who tested RT-PCR negative for SARS-CoV-2 at enrollment and had serologic evidence of SARS-CoV-2 infection 28–42 days after illness onset

^2^ Participants who responded “yes” to the question “Have you fully or mostly recovered from your illness?” on the first follow-up survey 7–14 days after illness onset.

**Supplemental Figure 1.** Survey completion and participant exclusions.

Initiated 2^nd^ follow-up survey (n=650)

Participants excluded:

Incomplete survey data (n = 30)

Vaccinated prior to the enrollment illness onset (n =23)

Uninterpretable SARS-CoV-2 test result (n =8)

Asymptomatic at enrollment (n= 7)

Tested >10 days after illness onset (n= 4)

Included in analyses

(n = 578)

**Supplemental Figure 2.** Adjusted Global Health, Physical Function, Fatigue, and Dyspnea domain mean T-score ratios from multivariable^1-3^ Gamma regression models.


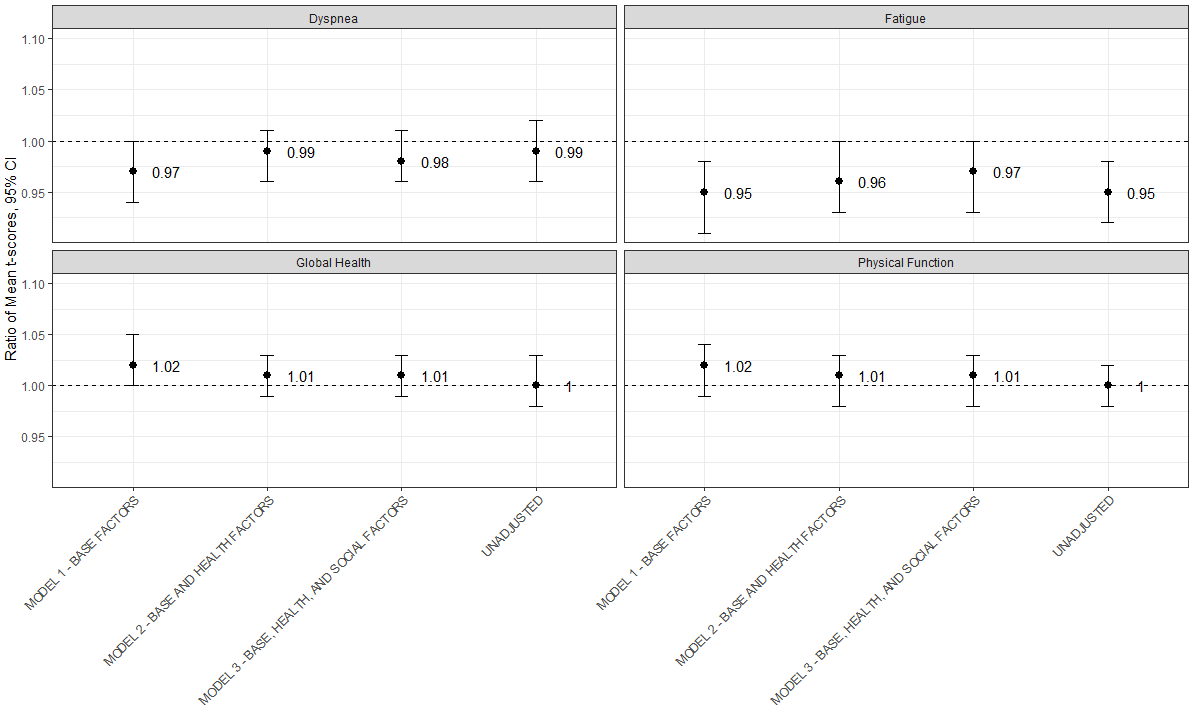


^1^ Base model includes adjustment for participant age, sex, interval between onset and follow-up survey completion, and study site.

^2^ Health factor models include adjustment for factors in the base model plus presence of any underlying health condition, self-reported cigarette smoking, body mass index, and self-rated general health status.

^3^ Social factor models include adjustment for factors in the base model plus self-reported race and Hispanic ethnicity, education level, and seasonal influenza vaccination status
